# Mediation of Internet addiction on association between childhood maltreatment and suicidal behaviours among Chinese adolescents

**DOI:** 10.1017/S2045796021000524

**Published:** 2021-10-11

**Authors:** Chang Peng, Mengni Wang, Junhan Cheng, Yafei Tan, Yunxiang Huang, Fajuan Rong, Chun Kang, Hongli Ding, Yan Wang, Yizhen Yu

**Affiliations:** 1 Department of Maternal, Child and Adolescent Health, School of Public Health, Tongji Medical College, Huazhong University of Science and Technology, Wuhan 430030, Hubei, China; 2 Wuhan Children's Hospital, Tongji Medical College, Huazhong University of Science and Technology, Wuhan 430016, Hubei, China; 3 Chinese Evidence-based Medicine Center, West China Hospital, Sichuan University, Chengdu 610041, Sichuan, China

**Keywords:** Child abuse, child psychiatry, maltreatment, mental health, suicide

## Abstract

**Aims:**

The associations between suicidal behaviours and childhood maltreatment (CM), as well as Internet addiction (IA) have been extensively examined. However, few studies pay attention to different types of CM and all stages of suicidality, including suicidal ideation (SI), suicidal plans (SP) and suicidal attempts (SA). Moreover, little is known regarding the mediation of IA on the relationship between CM and suicidal behaviours. The study aims to explore the direct effect of CM and IA on three stages of suicidal behaviours, and the indirect effect of CM on suicidality via IA.

**Methods:**

A total of 16 130 high-school students aged 12–18 were recruited using a stratified cluster sampling strategy across five representative provinces in China. Relevant information was collected by a self-administered anonymous questionnaire. Multinomial logistic regression analysis and structural equation model were used to examine the associations.

**Results:**

During the last year, 16.0% of participants reported suicidal behaviours. Specifically, 7.9% reported SI only, 4.6% reported SP but no SA, and 3.5% reported SA. The prevalence of neglect, physical abuse and IA in moderate to severe were 28.9, 19.9 and 33.1%, respectively. After controlling for demographic characteristics and confounding factors, such as loneliness, psychological resilience, and social support, moderate and severe neglect, physical abuse and IA were associated with an increased risk of SI, SP and SA (*p* < 0.01). The total effect of neglect and physical abuse on suicidal behaviours were 0.152 and 0.172, respectively (*p* < 0.001). The mediation proportion of IA on the association between neglect and suicidal behaviours, as well as physical abuse and suicidal behaviours were 22.4 and 18.0%, respectively.

**Conclusions:**

CM and IA are independently associated with suicidal behaviours among Chinese adolescents. Moreover, IA plays a mediating role on the relationship between CM and suicidality. Targeted interventions for adolescents’ suicidal behaviours should focus on those who have experience of CM and IA.

## Introduction

About 800–000 people die from suicide each year and 78% of these suicides occur in low- and middle-income countries (World Health Organization (WHO), 2019). Suicide can occur in all populations and of all ages. For adolescents aged 15–29 years, suicide is the second cause of death (WHO, 2020). Indeed, the rates of young people losing their lives to suicide have grown in recent years, especially in developing countries (Qu *et al*., 2021). Therefore, it is great importance to pay more attention to suicidal behaviours among adolescents (Shen *et al*., 2020).

It is widely accepted that suicidal behaviours were a continuum of development from mild to severe, most often including the following stages: suicidal ideation (SI), suicidal plans (SP), suicidal attempts (SA) and suicide death (Sveticic and De Leo, 2012; Qu *et al*., 2021). SI concerns thoughts about ending one's own life (suicidal thoughts). SP involves making plans about how to kill oneself. SA are the actions undertaken to complete suicide (Karanikola *et al*., 2019). The estimated worldwide lifetime prevalence for SI ranged from 12.1 to 31.5% and that for SA ranged from 4.1 to 23.5% (Chiu *et al*., 2018). Compared to SI and SA, researchers pay less attention to SP (Angelakis *et al*., 2020; Shen *et al*., 2020). Indeed, it is essential for suicide prevention to comprehensively understand the whole suicidal model, including all SI, SP and SA. From this point of view, it seems inappropriate to dichotomise any stage of suicidal behaviours (Sveticic and De Leo, 2012). Alternatively, individuals with or without suicidal behaviours should be classified into four groups: None group (without SI, SP or SA), SI group (have SI only, neither SP nor SA), SP group (have SP, but no SA) and SA group (have SA) (Sveticic and De Leo, 2012).

Multiple factors have been examined to be associated with suicidal behaviours, including genetic, psychological and social factors (Liu *et al*., 2019; Ati *et al*., 2021; Qu *et al*., 2021). Previous studies have documented that childhood maltreatment (CM) is a significant risk factor for suicidal behaviours (Miller *et al*., 2013; Behr Gomes Jardim *et al*., 2018; Angelakis *et al*., 2019, 2020). CM refers to acts of commission or omission by a parent or other caregiver that results in actual harms or threats of harm to a child under age 18, even if the harm is unintentional (Falgares *et al*., 2018). In China, a systematic review estimated that the prevalence of core forms of CM among adolescents from high to low was physical abuse (26.6%), neglect (26.0%), emotional abuse (19.6%) and sexual abuse (8.7%) (Fang *et al*., 2015). Recently, scholars have made efforts to assess the specific impact of different forms of CM on suicidal risk (Falgares *et al*., 2018; Angelakis *et al*., 2020). Therefore, it is necessary to primarily examine the association between two of the most prevalent forms of CM and suicidal behaviours among Chinese adolescents, which included neglect and physical abuse (Wang *et al*., 2020).

In addition to CM, a lot of research indicated that Internet addiction (IA) was also significantly associated with suicidal risk and the participants with IA generally have higher rates of suicidal behaviours (Kim *et al*., 2017; Liu *et al*., 2017; Huang *et al*., 2020; Shen *et al*., 2021). IA is characterised by preoccupation with the use of the Internet, failure to control the desire to access the Internet and continuous use of the Internet despite varying levels of functional impairment (Kuss *et al*., 2014; Shen *et al*., 2021). Research evidence supports that, compared with adults, children and adolescents are more likely to be affected by IA because of their relatively low psychological maturity and susceptibility to emotionally charged situations (Arrivillaga *et al*., 2020; Shen *et al*., 2020). In the recent years, prior studies have reported an increasing prevalence of IA in adolescents worldwide (Cheng and Li, 2014; Li *et al*., 2018). In China, existing literature reported that the prevalence of IA among adolescents ranged from 13.5 to 31.0% (Wu *et al*., 2013; Guo *et al*., 2018*a*; Xin *et al*., 2018). However, up to now, most of the previous studies focus on college students, while evidence regarding the prevalence of IA based on high school students is scant (Li *et al*., 2018; Shen *et al*., 2020, 2021). What's more, few studies have revealed the relationships between IA and all three stages of suicidal behaviours (Guo *et al*., 2018*a*; Arrivillaga *et al*., 2020; Shen *et al*., 2020). Therefore, the associations between IA and SI, SP and SA among high school students need to be further unravelled (Ko *et al*., 2009), which is important for suicide prevention (Sun *et al*., 2019).

Furthermore, a growing number of studies have provided evidence to support that maltreated adolescents were more likely to have IA than those who have no history of CM (Hsieh *et al*., 2016; Lo *et al*., 2021). To sum up, although previous studies have demonstrated the association between CM and IA, IA and suicidal behaviours, as well as CM and suicidal behaviours, few studies have revealed the relationship among three of them (Falgares *et al*., 2018; Lu *et al*., 2020). To our knowledge, there is only one study exploring the mediating role of IA on the association between childhood trauma and SI in Chinese population. Unfortunately, the study failed to reveal the correlation between different types of CM and three stages of suicidal behaviours (Lu *et al*., 2020). Hence, it has great significance to examine further the underlying mediation of IA on the relation between CM and suicidal behaviours. If a positive relationship does exist among them, it will help scholars and educators to better understand the development and mechanism of suicidal behaviours among adolescents. At the same time, it is meaningful for public policy makers to implement suicidal prevention and intervention.

To fill the gap, the primary purpose of the study is to examine the direct association between different types of CM (including neglect and physical abuse), IA and suicidal behaviours (including SI, SP and SA). We hypothesised that neglect, physical abuse and IA are independently associated with SI, SP and SA after adjusted for demographic characteristics and some potential confounders such as loneliness, psychological resilience, social support (Shen *et al*., 2020; Lo *et al*., 2021). The secondary objective is to explore the mediating role of IA in the relationship between CM and suicidal behaviours using structural equation modelling. We hypothesised that neglect and physical abuse would indirectly related to suicidal behaviours via IA (Lu *et al*., 2020).

## Methods

### Study design and data collection

A multi-stage cluster sampling was adopted in this cross-sectional study from February to October in 2015. In stage 1, China was divided into five regions by geographic locations (northern, southern, eastern, western and central part). Five representative provinces (Heilongjiang, Guangdong, Anhui, Yunnan and Hubei) were randomly selected from each region (Tang *et al*., 2020). In stage 2, three counties were chosen randomly in each province. In stage 3, with the help of local educational bureaus, we selected two or three high schools in each county based on enrolment size. In stage 4, in each selected school, we used random digits to choose two or three classes from each grade (from 7th to 12th) with the help of school administrators. Finally, we invited all students in the selected class to participate in this survey voluntarily.

All participants were informed that they had the right to withdraw at any time. Then, they were explicitly assured that all responses in the survey would be treated confidentially and anonymously. Informed written consent was obtained from each participant and their parents (or legal guardians) before the filed investigation. The study was approved by the Medical Ethics Committee of Tongji Medical College, Huazhong University of Science and Technology. We followed the Strengthening the Reporting of Observational Studies in Epidemiology (STROBE) guidelines.

Excluding those who refused to participate in the study, a total of 16 400 students were recruited in our study. Among the respondents, 270 were excluded due to the missing data was more than 15% of items on the whole questionnaire. Finally, 16 130 students’ questionnaires were qualified and the actual response rate was 98.35% (16 130/16 400).

## Assessment

### Suicidal behaviours

Suicidal behaviours include SI, SP and SA. In this study, SI, SP and SA were measured by the following questions during the past year (Wan *et al*., 2019). SI: ‘Have you ever had thoughts of committing suicide?’ SP: ‘Have you ever made a specific plan about how you would kill yourself?’ and SA: ‘Have you ever tried to commit suicide?’ The response was yes or no (Shen *et al*., 2020). Participants were considered to have experience of SI, SP or SA if the response was yes (Posner *et al*., 2007; Zhang *et al*., 2018).

Since some participants would have simultaneously experienced SI, SP and SA, we classified participants into four categories in the current study: 0 = None group (without SI, SP or SA), 1 = SI group (have SI only, neither SP nor SA), 2 = SP group (have SP, but no SA), 3 = SA group (have SA) (Sveticic and De Leo, 2012).

### Neglect and physical abuse

Neglect and physical abuse were measured by two subscales from the Parents – Child Conflict Tactics Scale (CTS – PC) (Straus *et al*., 1998). Using subscales from CTS – PC has been employed in a previous study (Chan, 2015). The tool was translated into Chinese and has demonstrated its ability to identify CM (Chan *et al*., 2011; Lo *et al*., 2021). The neglect subscale contains five items and the physical abuse subscale comprises 12 items, including four items for mild physical abuse, four items for moderate physical abuse and four items for severe physical abuse. The participants were asked how frequently they had encountered the listed behaviours in the past year using a three-point Likert scale: 0 = none, 1 = once and 2 = twice or more. The total score of the neglect subscale ranges from 0 to 10. We defined the total score of 0 (none), 1–3 (mild), 4–6 (moderate) and 7–10 (severe) as four categories of neglect (Chang *et al*., 2017). We defined the participants who did not experience any behaviours from physical abuse subscales as none physical abuse. Participants who only experienced behaviours from the dimension of mild physical abuse were defined as mild physical abuse. Participants who experienced any behaviours from the dimension of moderate physical abuse but did not experience any behaviours from the dimension of severe physical abuse were defined as moderate physical abuse. Participants who experienced any behaviours from the dimension of severe physical abuse were defined as severe physical abuse (Chang *et al*., 2017). The Cronbach's *α* of the whole scale, neglect and physical abuse subscales were 0.798, 0.721 and 0.807, respectively.

### Internet addiction

IA was assessed using the Chinese version of the Young's Internet addiction test (IAT) that has been validated in Chinese adolescents with satisfactory psychometric properties (the Cronbach's *α* was 0.93) (Lai *et al*., 2013; Lo *et al*., 2021). The IAT comprises 20 items rated in a 5-point Likert scale (from 1 = ‘not at all’ to 5 = ‘always’). The total score of the IAT ranges from 20 to 100. The higher score suggests the greater level of an individual's tendency to IA (Young, 1998). In this study, we not only described the total IAT scores but also used the validated standard cut-off criteria, which including ‘average online users’ (20–49 points), ‘moderate IA’ (50–79 points) and ‘severe IA’ (80–100 points) (Young and de Abreu, 2011). Participants were considered to have IA if they were classified as ‘moderate IA’ or ‘severe IA’ (Lu *et al*., 2020). The Cronbach's *α* of the IAT in this study was 0.933.

### Confounding and demographic variables

Confounding variables included loneliness, psychological resilience and social support. Detailed information about confounding and demographic variables is presented in supplement.

## Data analyses

First, categorical variables were summarised by prevalence or proportion [*n* (%)], while continuous variables were described by mean (s.d.). Second, the univariate analysis of suicidal behaviours was assessed using the chi-squared test and ANOVA. Then, in order to examine the independent effects of neglect, physical abuse and IA on suicidal behaviours, a model of multinomial logistic regression analysis was performed after controlling for demographic characteristics, loneliness, psychological resilience and social support. The dependant variable was suicidal behaviours (0 = None group, 1 = Suicidal ideation group, 2 = Suicidal plans group, 3 = Suicidal attempts group) as mentioned above in Assessment. The independent variables were neglect (0 = None, 1 = Mild, 2 = Moderate, 3 = Severe), physical abuse (0 = None, 1 = Mild, 2 = Moderate, 3 = Severe) and IA (0 = None, 1 = Moderate, 2 = Severe). We included gender, age, single-child family, family composition, caregiver, caregiver's education, family income, loneliness score, psychological resilience score and social support score as confounding variables. The results were displayed with odds ratios (ORs) and 95% confidence intervals (95% CIs). The threshold of significance was defined as *p* < 0.05. All statistical analyses were performed using SPSS 26.0.

We performed a set of structural equation model (SEM) to evaluate the mediating effects of IA in the relationship between CM and suicidal behaviours. Neglect and physical abuse were included in SEM separately. In model 1, we ran SEM without demographic characteristics and confounders. In model 2, we ran SEM again after adjusted variables that showed statistical significance in multinomial logistic regression analysis. All analyses of SEM were performed using AMOS 21.0.

## Results

### Sample characteristics

We included 16 130 participants in the final analyses. The proportion of males was slightly higher than females (51.9% *v*. 48.1%). The age of the participants ranged from 12 to 18 years; the mean (s.d.) age was 15.22 (1.79) years. Other demographic characteristics of participants are reported in Table 1.

**Table 1.** Basic characteristics of participants and the prevalence of suicidal behaviours

Variables	Total	Suicidal behaviours				*χ*^2^/*F*
		None	Suicidal ideation	Suicidal plans	Suicidal attempts	
*Categorical variables*						
Gender^a^						26.31***
Boy	8368 (51.9)	7147 (85.4)	587 (7.0)	359 (4.3)	275 (3.3)	
Girl	7762 (48.1)	6404 (82.5)	680 (8.8)	384 (4.9)	294 (3.8)	
Single-child family^a^						3.97
Yes	5564 (34.7)	4671 (84.0)	427 (7.7)	279 (5.0)	187 (3.4)	
No	10458 (65.3)	8788 (84.0)	834 (8.0)	460 (4.4)	376 (3.6)	
Family composition^a^						42.38***
Two biological parents	14 128 (89.5)	11 939 (84.5)	1095 (7.8)	619 (4.4)	475 (3.4)	
Single biological parent	1156 (7.3)	911 (78.8)	114 (9.9)	76 (6.6)	55 (4.8)	
Others	499 (3.2)	396 (79.4)	39 (7.8)	38 (7.6)	26 (5.2)	
Caregiver^a^						18.16***
Parents	12 197 (76.9)	10 318 (84.6)	936 (7.7)	544 (4.5)	399 (3.3)	
Grandparents	2036 (12.8)	1678 (82.4)	171 (8.4)	101 (5.0)	86 (4.2)	
Others	1620 (10.2)	1325 (81.8)	131 (8.1)	88 (5.4)	76 (4.7)	
Caregiver's education^a^						36.11***
Primary school or less	4342 (27.6)	3636 (83.7)	371 (8.5)	192 (4.4)	143 (3.3)	
Junior high school	6985 (44.4)	5932 (84.9)	486 (7.0)	325 (4.7)	242 (3.5)	
Senior high school	3356 (21.3)	2798 (83.4)	289 (8.6)	160 (4.8)	109 (3.2)	
College or more	1059 (6.7)	854 (80.6)	88 (8.3)	53 (5.0)	64 (6.0)	
Family income (RMB)^a^						43.69***
6000−	2136 (14.0)	1745 (81.7)	175 (8.2)	113 (5.3)	103 (4.8)	
4000−5999	2777 (18.2)	2327 (83.8)	234 (8.4)	113 (4.1)	103 (3.7)	
2000−3999	5682 (37.2)	4809 (84.6)	451 (7.9)	256 (4.5)	166 (2.9)	
1000−1999	2646 (17.3)	2264 (85.6)	192 (7.3)	111 (4.2)	79 (3.0)	
−999	2021 (13.2)	1654 (81.8)	154 (7.6)	118 (5.8)	95 (4.7)	
Neglect						633.04***
None	5336 (33.1)	4826 (90.4)	276 (5.2)	145 (2.7)	89 (1.7)	
Mild	6143 (38.1)	5282 (86.0)	440 (7.2)	243 (4.0)	178 (2.9)	
Moderate	3190 (19.8)	2461 (77.1)	350 (11.0)	210 (6.6)	169 (5.3)	
Severe	1461 (9.1)	982 (67.2)	201 (13.8)	145 (9.9)	133 (9.1)	
Physical abuse						603.04***
None	10 784 (66.9)	8443 (87.6)	713 (6.6)	384 (3.6)	244 (2.3)	
Mild	2148 (13.3)	1787 (83.2)	182 (8.5)	103 (4.8)	76 (3.5)	
Moderate	2493 (15.5)	1880 (75.4)	283 (11.4)	183 (7.3)	147 (5.9)	
Severe	705 (4.4)	441 (62.6)	89 (12.6)	73 (10.4)	102 (14.5)	
Internet addiction						683.43***
None	10 799 (66.9)	9562 (88.5)	672 (6.2)	330 (3.1)	235 (2.2)	
Moderate	4804 (29.8)	3679 (76.6)	521 (10.8)	340 (7.1)	264 (5.5)	
Severe	527 (3.3)	310 (58.8)	74 (14.0)	73 (13.9)	70 (13.3)	
Total	16 130 (100.0)	13 551 (84.0)	1267 (7.9)	743 (4.6)	569 (3.5)	
*Continuous variables*						
Age, mean (s.d.)	15.22 (1.79)	15.26 (1.80)	15.13 (1.77)	15.03 (1.69)	14.86 (1.55)	13.32***
Loneliness, mean (s.d.)	44.28 (14.09)	42.95 (13.30)	50.28 (15.66)	51.95 (15.74)	52.60 (16.63)	269.20***
Psychological resilience, mean (s.d.)	91.43 (13.17)	92.58 (13.00)	86.61 (12.15)	84.70 (12.32)	83.49 (12.55)	234.79***
Social support, mean (s.d.)	62.80 (14.39)	64.07 (13.93)	57.76 (13.91)	54.36 (15.10)	54.81 (16.37)	240.97***

a There was missing data (single-child family = 108, family composition = 347, caregiver = 277, caregiver's education = 388, family income = 868).

***p < 0.001.

Of the participants, 16.0% of them reported suicidal behaviours during the last year. Specifically, 7.9% reported SI only, neither SP nor SA; 4.6% reported SP, but no SA; 3.5% reported SA, regardless of SI or SP. About one-fifth (19.9%) of participants reported moderate-to-severe physical abuse and 28.9% reported moderate to severe neglect in the past year. Near one-third (33.1%) of participants reported moderate-to-severe IA (Table 1).

### Multinomial logistic regression analyses

Univariate analysis of suicidal behaviours is also displayed in Table 1. After testing the normality of independent variables, dependant variables and some confounders, Spearman's correlations are shown in Table 2. Multinomial logistic regression analysis of suicidal behaviours is shown in Table 3. After controlling for demographic characteristics and confounding factors, mild, moderate and severe neglect were all associated with an increased risk of SI, SP and SA, except for the association between mild neglect and SP. Compared to those who reported none physical abuse, participants who experienced moderate and severe physical abuse had greater odds of SI, SP and SA (all *p* < 0.01). In addition, moderate and severe IA was associated with higher risk of SI, SP and SA (all *p* < 0.001).

**Table 2.** Correlations among independent variables, dependant variables and some confounders

Variables	1	2	3	4	5	6	7
1. Neglect	–						
2. Physical abuse	0.305**	–					
3. Internet addiction	0.205**	0.155**	–				
4. Loneliness	0.2.58**	0.156**	0.249**	–			
5. Psychological resilience	−0.233**	−0.196**	−0.252**	−0.488**	–		
6. Social support	−0.251**	−0.159**	−0.275**	−0.546**	0.482**	–	
7. Suicidal behaviours	0.188**	0.163**	0.206**	0.198**	−0.202**	−0.198**	–

**p < 0.01.

**Table 3.** Multinomial logistic regression of suicidal behaviours [OR (95% CI)]^a^

Variables	Suicidal ideation	Suicidal plans	Suicidal attempts
Neglect (ref. = none)			
Mild	1.21 (1.02–1.43)*	1.18 (0.94–1.48)	1.35 (1.02–1.77)*
Moderate	1.66 (1.38–2.00)***	1.66 (1.31–2.11)***	1.89 (1.41–2.52)***
Severe	1.86 (1.49–2.33)***	2.11 (1.60–2.78)***	2.90 (2.10–4.00)***
Physical abuse (ref. = none)			
Mild	1.13 (0.94–1.36)	1.13 (0.89–1.44)	1.30 (0.98–1.72)
Moderate	1.44 (1.22–1.69)***	1.61 (1.32–1.98)***	1.85 (1.46–2.34)***
Severe	1.48 (1.14–1.93)**	1.83 (1.36–2.48)***	3.59 (2.69–4.81)***
Internet addiction (ref. = none)			
Moderate	1.59 (1.39–1.81)***	2.01 (1.69–2.39)***	2.13 (1.74–2.60)***
Severe	2.15 (1.61–2.88)***	3.89 (2.86–5.29)***	4.77 (3.43–6.64)***
Loneliness	1.02 (1.01–1.02)***	1.02 (1.01–1.02)***	1.02 (1.01–1.02)***
Psychological resilience	0.99 (0.98–0.99)***	0.98 (0.97–0.99)***	0.98 (0.97–0.99)***
Social support	0.99 (0.98–0.99)**	0.98 (0.97–0.99)***	0.99 (0.98–0.99)**
Gender (ref. = male)	1.58 (1.39–1.80)***	1.66 (1.41–1.96)***	1.61 (1.33–1.94)***
Age	0.95 (0.92–0.99)**	0.92 (0.87–0.96)***	0.88 (0.83–0.93)***
Single-child family (ref. = no)	1.05 (0.91–1.12)	1.22 (1.03–1.45)*	0.96 (0.78–1.18)
Family composition (ref. = two biological parents)			
Single biological parent	1.18 (0.94–1.47)	1.32 (1.01–1.72)*	1.19 (0.87–1.63)
Others	1.00 (0.69–1.46)	1.42 (0.92–2.18)	1.32 (0.81–2.13)
Caregiver (ref. = parents)			
Grandparents	0.95 (0.77–1.16)	0.90 (0.69–1.18)	1.06 (0.79–1.43)
Others	0.93 (0.75–1.15)	1.05 (0.80–1.38)	1.34 (0.99–1.80)
Caregiver's education (ref. = primary school or less)			
Junior high school	0.90 (0.77–1.05)	1.17 (0.96–1.44)	1.20 (0.95–1.51)
Senior high school	1.12 (0.93–1.35)	1.21 (0.95–1.55)	1.12 (0.84–1.50)
College or more	1.19 (0.91–1.55)	1.34 (0.95–1.90)	2.32 (1.63–3.30)***
Family income (RMB) (ref. = 6000~)			
4000−5999	0.98 (0.79–1.21)	0.74 (0.56–0.98)*	0.81 (0.60–1.09)
2000−3999	0.88 (0.73–1.07)	0.78 (0.62–0.99)*	0.62 (0.47–0.82)**
1000−1999	0.76 (0.61–0.96)*	0.71 (0.53–0.94)*	0.62 (0.45–0.85)**
−999	0.84 (0.66–1.07)	1.09 (0.82–1.45)	1.05 (0.77–1.44)

a The reference category for the dependant variables were none (without SI, SP or SA).

***p < 0.001, **p < 0.01, *p < 0.05.

Besides, loneliness was positively while psychological resilience and social support were negatively associated with SI, SP and SA. Compared to the boy, the girl had higher odds of SI, SP and SA (all *p* < 0.01). Moreover, age was negatively associated with all SI, SP and SA (Table 3).

### Structural equation modelling

**Fig. 1.**
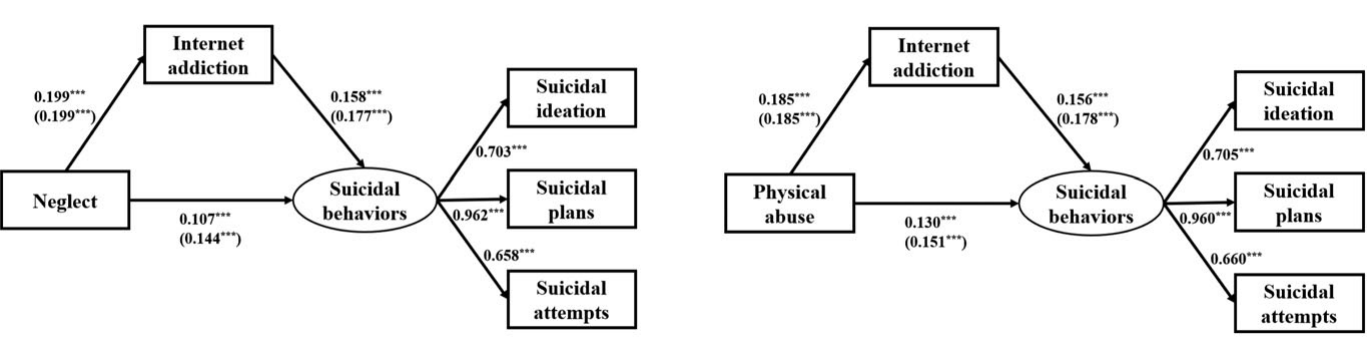
Structural equation modelling depicting direct effects of neglect, physical abuse and IA on suicidal behaviours. Adjusted *β* coefficients in model 2 (unadjusted *β* coefficients in model 1) are presented. Model 1: Without adjusted for confounders. Model 2: Adjusted for age, gender, loneliness, psychological resilience and social support. ***p < 0.001.

Figure 1 shows the results of SEM. After controlling for those variables that significantly associated with all SI, SP and SA in multinomial logistic regression analysis, there were direct effects of neglect (*β* = 0.107, *p* < 0.001) and IA (*β* = 0.158, *p* < 0.001) on suicidal behaviours. The total effect of neglect on suicidal behaviours was 0.139 (*p* < 0.001). The mediating effect of IA was 0.031 (*p* < 0.001). The mediation proportion was 22.3%. Similarly, there were direct effects of physical abuse (*β* = 0.130, *p* < 0.001) and IA (*β* = 0.156, *p* < 0.001) on suicidal behaviours. The total effect of physical abuse on suicidal behaviours was 0.159 (*p* < 0.001). The mediating effect of IA was 0.029 (*p* < 0.001). The mediation proportion was 18.2% (Table 4). Goodness-of-fit indices (i.e. CFI > 0.900, TLI > 0.900, RMSEA < 0.05, SRMR < 0.05) indicated satisfactory fit of all models of the SEM.

**Table 4.** Mediating effect of IA between CM and suicidal behaviours [*β* (95% CI)]

Variables	Neglect		Physical abuse	
	Model 1	Model 2	Model 1	Model 2
Childhood maltreatment → suicidal behaviours	0.144 (0.125–0.163)***	0.107 (0.088–0.127)***	0.151 (0.128–0.174)***	0.130 (0.107–0.154)***
Internet addiction → suicidal behaviours	0.177 (0.157–0.197)***	0.158 (0.137–0.180)***	0.178 (0.158–0.198)***	0.156 (0.134–0.178)***
Childhood maltreatment → Internet addiction	0.199 (0.182–0.215) ***	0.199 (0.182–0.215)***	0.185 (0.167–0.204)***	0.185 (0.167–0.204)***
Standardised effect				
Indirect	0.035 (0.030–0.041)***	0.031 (0.026–0.037)***	0.033 (0.028–0.038)***	0.029 (0.024–0.034)***
Total	0.179 (0.160–0.198)***	0.139 (0.118–0.159)***	0.184 (0.161–0.206)***	0.159 (0.136–0.183)***
Mediating ratio, %	19.6	22.3	17.9	18.2

Model 1: Without adjusted for confounders.

Model 2: Adjusted for age, gender, loneliness, psychological resilience and social support.

***p < 0.001.

## Discussion

This is the first study to explore the mediation of IA on the association between different types of CM and suicidal behaviours. There were two key and novel findings from this study. First, neglect, physical abuse and IA were independently associated with SI, SP and SA after controlling for loneliness, psychological resilience, social support and some demographic variables. Second, there were indirect effects of neglect and physical abuse on suicidal behaviours, partially mediated by IA. These findings provide new information about the relationship between CM, IA and suicidal behaviours among adolescents, which could benefit for educators, scholars and decision-makers to better understand the development of adolescents’ suicidal behaviours.

Compared to adolescents with no history of CM, those adolescents who experienced moderate and severe neglect, as well as physical abuse, were more likely to report all of SI, SP and SA. The finding of this study is in accordance with most of previous works (Guo *et al*., 2018*b*; McMahon *et al*., 2018; Lu *et al*., 2020). According to a newly published systematic review with a total number of 79 studies performed from 1989 to 2019, physical abuse was associated with 1.95 times in the odds for SI and 2.18 times for SA among adolescents with the average age was 15.67 (2.11) years. In addition, neglect, including physical and emotional neglect, was associated with an over 1.5-fold increase in the odds for SA (Angelakis *et al*., 2020). Unfortunately, this review failed to reveal the association between neglect and SI, as well as SP. Therefore, more research exploring the relationships between different types of CM and three stage of suicidal behaviours needs to be undertaken (Angelakis *et al*., 2020).

The use of the Internet for social networking and learning is increasing, especially for children and adolescents (Arrivillaga *et al*., 2020). At the extreme end, they could become dependent on the Internet and are prone to IA (Lo *et al*., 2021). A bulk of prior studies have examined the potentially effects of IA on adolescent's mental and conduct disorders, such as sleep disturbances, depression, anxiety, aggression and attention deficit hyperactivity disorder (ADHD) (Ferrara *et al*., 2017; Kim *et al*., 2018; SeungYup *et al*., 2018; Tang *et al*., 2020; Lo *et al*., 2021). In the current study, we found that IA significantly increased the risk of all SI, SP and SA among adolescents. This finding is aligns with most recent previous studies, which support a positive correlation between IA and suicidal behaviours (Lin *et al*., 2014; Marchant et al., 2017; Guo *et al*., 2018*a*; Pan and Yeh, 2018). However, another survey based on 8130 college students in Hunan Province of China indicated that SI and SA were independent correlates for IA after controlling for depression and anxiety, while the association between IA and SP has no statistical significance (Shen *et al*., 2020). Therefore, more research should be conduct to examine further the relationship between IA and three stages of suicidal behaviours, including SI, SP and SA.

Beyond the direct effects of CM and IA on suicidal risk, we found that IA also played a mediating role in the relationship between CM and suicidal behaviours. In other words, adolescents with suicidal behaviours may overuse Internet as a coping strategy to alleviate stressful feelings from CM (Guo *et al*., 2018*a*). Several possible reasons could partly explain the phenomenon. One the one hand, family is an important source of security and support, while CM can impair adolescents’ perception of family support. Then, it could, in turn, increase their usage of the Internet to escape from stressful family environments, which further increase the risk for IA. In response to CM and inadequate family support, IA is a maladaptive form of avoidance coping strategy and a self-medicating behaviour (Hsieh *et al*., 2016). Therefore, maltreated adolescents growing up in a dysfunctional family environment are more likely to perceive less support from the family and develop poor interpersonal relationships in the real world. This may, in turn, increase the adolescents’ risk for IA (Wu *et al*., 2016; Wang and Qi, 2017; Alto *et al*., 2018).

On the other hand, the anonymous nature of the Internet may provide an alternative safe room for maltreated adolescents to cope with the adverse childhood experiences by burying them in the Internet-based social network. Maltreated adolescents may perceive the virtual world as a security environment that allows them to express their stressful feelings. In addition, playing and interacting with other people online seems to help them to develop a better sense of relatedness and competence (Lo *et al*., 2021). However, instead of reducing the adverse affect of CM, they may become increasingly reliant on IA as a negative coping tool to avoid stressful feelings (Lo *et al*., 2021). Consequently, CM can be indirectly linked to suicidal behaviours through IA (Lu *et al*., 2020).

### Limitations

Several limitations should be noted. First, the cross-sectional study design makes it impossible to obtain the causal relationship of CM, IA and suicidal behaviours. A prospective longitudinal study is a benefit for clarifying this issue in the future research. Second, all of the variables were assessed by a self-reported questionnaire, which may augment underestimation of some sensitive issues regarding to CM, IA, as well as suicidal behaviours and increase potential reporting bias and recall bias. In particular, three stages of suicidal behaviours were assessed with single items instead of dedicated interviews or psychometric instruments. Although the measurement of this study is practical and efficient in a large-size sample study (Guo *et al*., 2021), we will adopt more rigorous assessment in future studies. Third, we only included two core subtypes of CM in the analysis, including neglect and physical abuse, while the direct and indirect influence of emotional abuse and sexual abuse on suicidal risk was ignored. However, a recent systematic review provided a quantifiable evidence to support a strong association between sexual abuse and all three stages of suicidal behaviours (Angelakis *et al*., 2020). Hence, in our future work, we will include all core forms of CM and further explore which form has the strongest predictive effect for IA and suicidal behaviours among Chinese adolescents. Finally, although this study achieved a large sample size across China, the participants were recruited only from rural areas, limiting the generalisability of our findings for whole Chinese adolescents. Therefore, our future research is supposed to recruit more participants through a multi-centre sampling method.

## Conclusion

Neglect, physical abuse and IA are significantly associated with three stages of suicidal behaviours among Chinese adolescents, including SI, SP and SA. Moreover, IA plays a mediating role in the relationship between neglect, physical abuse and suicidal behaviours. These findings extended existing literature by first exploring the relationship of different types of CM, IA and all stages of suicidal behaviours. Targeted interventions for adolescents’ suicidal behaviours should focus on those who experienced CM and IA.

## Data Availability

The data of the current study is available from the corresponding author.

## Supplementary material

For supplementary material accompanying this paper visit http://dx.doi.org/10.1017/S2045796021000524. click here to view supplementary material
