# Supplementary material for: Mediation of Internet addiction on association between childhood maltreatment and suicidal behaviours among Chinese adolescents
Source: Epidemiol Psychiatr Sci. 2021 Oct 11;30:e64. doi: 10.1017/S2045796021000524 (PMC8518027; doi:10.1017/S2045796021000524)
Supplement: Supplementary file 1 [file S2045796021000524sup001.docx]

*Confounding variables*

Loneliness was measured by the University of California Los Angeles Loneliness Scale (UCLALS). The UCLALS was a unidimensional, self-report measure of perceived isolation and had 21 items with 5-point Likert scale responses (from 1 = “totally inconsistent” to 5 = “fully consistent”) (Russell, 1996). Higher total scores indicating greater loneliness. The UCLALS shows high internal consistency and adequate convergent validity (the Cronbach’s α was 0.94) (Hirsch *et al.*, 2012). The Cronbach’s α of the UCLALS in this study was 0.918.

Psychological resilience was measured using the Resilience Scale for Chinese Adolescents (RSCA). It has 27 items using a 5-point Likert scale (from 1 = “totally inconsistent” to 5 = “fully consistent”) (Hu, 2008). Higher total scores of RSCA indicating better psychological resilience. Previous studies have shown that the RSCA has good reliability and validity, and the Cronbach’s α of the scale was 0.90 (Han *et al.*, 2018). In the present study, the Cronbach’s α was 0.810.

Social support was measured by the Chinese version of the Adolescent Social Support Scale (ASSS), which has good reliability and validity to measure subjects’ individual differences in social resource utilisation (Dai, 2014). The ASSS has 17 items using a 5-point Likert scale (from 1 = “totally inconsistent” to 5 = “fully consistent”), with higher scores suggesting more perceived social support (Zhang *et al.*, 2018). The Cronbach’s α of the ASSS in this study was 0.939.

*Demographic variables*

Demographic variables included gender (1 = boy, 2 = girl), age, single‐child family (1 = yes, 0 = no), family composition (participant lives in a family with: 1 = two biological parents, 2 = a single biological parent, 3 = others) , caregiver (1= parents, 2 = grandparents, 3 = other), caregiver’s education (1 = primary school or less, 2 = junior high school, 3 = senior high school, 4 = college or more), and family income (average family income per month in RMB: 1 = 6000 ~ , 2 = 4000 ~ 5999, 3 = 2000 ~ 3999, 4 = 1000 ~ 1999, 5 = ~ 999).
